# Heart Failure 2012

**DOI:** 10.1155/2012/126324

**Published:** 2012-12-20

**Authors:** Gregory Giamouzis, George Giannakoulas, Javed Butler, John A. Elefteriades, Carsten Tschöpe, Filippos Triposkiadis

**Affiliations:** ^1^The University Hospital of Larissa, P.O. Box 1425, 41110 Larissa, Greece; ^2^A.H.E.P.A University Hospital, 54636 Thessaloniki, Greece; ^3^Emory University, Atlanta, GA 30322, USA; ^4^Yale University School of Medicine, New Haven, CT 06510, USA; ^5^Department of Cardiology and Pneumology, Charité-Universitätsmedizin Berlin, Campus Benjamin Franklin (CBF), Berlin, Germany

Heart failure has been named “the growing epidemic.” Over the last decade, the annual number of heart failure hospitalizations has almost doubled with approximately 50% of patients being rehospitalized within 6 months of discharge [1]. The complex array of physiologic, psychological, social, and health care delivery issues makes it a challenging chronic disease to manage. Understanding the epidemiology and pathophysiology of the syndrome, identifying the predictors and their strength of association with outcomes, and using the available diagnostic modalities cost-effectively are essential in order to devise effective prevention interventions and implement novel therapeutic approaches to curb this epidemic. 

In this special issue, we have invited a few papers that address such issues and explain why despite the emergence of novel therapeutic approaches, that promise life prolongation and hospital length reduction, this patient population will still be needing rehospitalization and will often have a poor prognosis. This special issue is the extension of an effort that was initiated in 2011 with the first heart-failure-focused issue [2].

 In the *pathophysiology section*, S. M. R. Kazemi-Bajestani et al. describe the opportunities and challenges of targeting the angiotensin converting enzyme 2 (ACE2)/Ang II/Ang1–7 and apelin/APJ pathways as novel therapeutic modalities in heart failure [3]. ACE2 and the apelin/APJ are two important peptide systems which exert diverse effects on the cardiovascular system. Dysregulation of such systems may be involved in the predisposition to cardiovascular diseases whereas enhancing their action may have important therapeutic effects. In the same section, D. Lindner et al. provide a comprehensive review on the protective function of signal transducer and activator of transcription 3 (STAT3) in CVB3-induced myocarditis [4]. The transcription factor (STAT3) is an important mediator of the inflammatory process, and in this original research the investigators examine the role of STAT3 in viral myocarditis and its possible role in the development to dilated cardiomyopathy.

 Considering the high mortality rate and the availability of life-saving therapies like transplantation and left ventricular assist devices, accurate *prognosis determination* in HF is clinically important. Taking into account the mediocre performance of current established prediction models, such as the Seattle Heart Failure Model [5–7], the work by H. Fukuta et al. [8] on the prognostic value of left ventricular diastolic dysfunction in patients undergoing cardiac catheterization for coronary artery disease sheds light on this extremely important topic.

A shared understanding of medical conditions between patients and their health care providers has been shown to improve self-care and outcomes [9]. In the *comorbidity section*, we demonstrate how certain comorbid conditions may affect patients' decision-making capacity and interfere with their ability to comply with treatment requirements, recognize and self-manage disease worsening symptoms. Among others, cognitive impairment is increasingly recognized as a common adverse consequence of HF, whereby phenomena such as microembolism, chronic or intermittent cerebral hypoperfusion, and/or impaired cerebral vessel reactivity may lead to cerebral hypoxia and ischemic brain damage. Cognitive decline in HF is characterized by deficits in one or more cognition domains, including attention, memory, executive function, and psychomotor speed. E. Dardiotis et al. [10], in a comprehensive review, underscore the importance for healthcare professionals to become familiar with assessment of cognitive performance using standardized screening instruments in their routine evaluations of HF patients. 

Another comorbidity gaining increasing attention in HF patients is depression. There are several pathophysiological mechanisms as well as behavioral processes linking depression and HF. Equally important is screening for depression and there are several valid and reliable screening tools to identify patients at greater risk. Consultation should be provided by a multidisciplinary team, consisting of cardiologists, psychiatrists, and hospital or community nurses so as to carefully plan, execute, and evaluate medical intervention and implement lifestyle changes. D. Mastrogiannis et al. [11] systematically review the existing knowledge regarding current definitions, prognostic implications, pathophysiological mechanisms, and current and future treatment options in patients with depression and HF. Evidence from the literature supports the possibility of a pathophysiological relationship between cognitive impairment, depression, and HF. Yet, very few studies have sought to investigate this relationship. The paper by Z. N. Sohani and Z. Samaan reviews current literature on the association between depression and cognitive impairment in persons with HF and explores possible mechanisms explaining this complex triad [12].

Heart failure through neurohumoral activation induces alterations of cardiac metabolism, such as insulin resistance, and promotes increased utilization of noncarbohydrate substrates for energy production [13, 14]. Fasting blood ketone bodies as well as fat oxidation have been shown to be increased in this patient population. The result is depletion of myocardial ATP, phosphocreatine, and creatine kinase, leading to decreased efficiency of mechanical work. A direct approach to manipulate cardiac energy metabolism consists in modifying substrate utilization by the failing heart. Trimetazidine, perhexiline, and ranolazine directly inhibit fatty acid oxidation and have been used to increase the ischemic threshold in patients with effort angina. Current research is supporting the concept that shifting the energy substrate preference away from fatty acid metabolism and toward glucose metabolism could be an effective adjunctive treatment in patients with HF. These agents have been shown to improve both glucose metabolism and left ventricular function in diabetic patients with left ventricular dysfunction. In the *pharmacotherapy section*, we provide a systematic review, in which N. Signoretta et al. [15] discuss the beneficial therapeutic effects of modulation of cardiac metabolic substrates utilization in patients with HF.

In the *advanced heart failure section*, we provide a thorough review on the current status of mechanical circulatory support in patients with advanced HF. Management of the advanced HF patients with the numerous comorbidities [16, 17] requires a significant amount of health care resources and is becoming a major public health problem. As therapeutical strategies for HF have been refined, the number of patients suffering from the end-stage disease has expanded dramatically. Although heart transplantation still represents the gold standard therapeutical approach, the shortage of donors universally has made the implantation of mechanical circulatory support devices a well-established management for this disease. The systematic review by K. Spiliopoulos et al. [18], outlines the current status of mechanical circulatory support in this patient population.

In the *chronic follow-up section*, we deal with telemonitoring, a novel diagnostic modality that has been suggested to be beneficial for HF patients, targeting optimization of their chronic followup. Telemonitoring is viewed as a means of recording physiological data (such as body weight, heart rate, arterial blood pressure electrocardiogram recordings, and other data) by portable devices and transmitting these data remotely (via a telephone line, a mobile phone, or a computer) to a server where they can be stored, reviewed, and analyzed by the research team. In a systematic review of all randomized clinical trials evaluating telemonitoring in chronic HF, G. Giamouzis et al. [19] assess whether telemonitoring provides any substantial benefit in this patient population. 

We hope that the readers of the journal will find the topics as interesting and important as we did.

*Gregory Giamouzis* *George Giannakoulas* *Javed Butler* *John A. Elefteriades* *Carsten Tschöpe* *Filippos Triposkiadis*
